# Volume electron microscopy reveals oligodendrocyte axon selection patterns and spatial assembly of myelin and nodes

**DOI:** 10.1016/j.isci.2026.117773

**Published:** 2026-10-07

**Authors:** Martina Monaco, Vanessa Chiappini, Maria Fernanda Veloz Castillo, Kalpana Kare, Marinella Clerico, Luca Bartesaghi, Corrado Calì

**Affiliations:** 1 Neuroscience Institute Cavalieri Ottolenghi, Regione Gonzole 10, 10043 Orbassano, Italy; 2 Department of Neuroscience “Rita Levi Montalcini”, University of Turin, 10124 Torino, Italy; 3 Carl Zeiss Microscopy, 73447 Oberkochen, Germany; 4 Biomedical Sciences Division, King Abdullah University of Science and Technology, Thuwal 23955, Saudi Arabia; 5 Biological and Environmental Sciences and Engineering Division, King Abdullah University of Science and Technology, Thuwal 23955, Saudi Arabia; 6 Department of Clinical and Biological Sciences, University of Turin, 10124 Torino, Italy; 7 Université Grenoble-Alpes, Fonds Clinatec, Grenoble, France

**Keywords:** oligodendrocyte, myelin, node of Ranvier, development, 3D reconstruction, serial block-face electron microscopy

## Abstract

Oligodendrocytes myelinate multiple axons, requiring single cells to coordinate target selection, sheath growth, and node formation across complex cortical geometry. We combined serial block-face electron microscopy, complete segmentation, and three-dimensional reconstruction to analyze early-myelinating oligodendrocytes in the somatosensory cortex of a postnatal day 14 rat at single-cell and nanoscale resolution. Individual oligodendrocytes initiated myelination across all contacted axons, while early ensheathment decreased with increasing process distance from the soma. Each oligodendrocyte also contacted axons within a restricted caliber range, independently of wrapping progression. At prospective nodes of Ranvier, transient cytoplasmic projections extended into axonal domains from adjacent oligodendrocytes and converged near presumptive paranodes. These observations support a structural model in which oligodendrocyte geometry, cell-specific axon selection, and intercellular interactions jointly organize early cortical myelination and nodal assembly, providing testable hypotheses for developmental myelination and repair after demyelination.

## Highlights

• Single oligodendrocytes preferentially contact axons within restricted caliber ranges
• Early myelin ensheathment decreases with oligodendrocyte process length
• Adjacent oligodendrocytes form transient projections at prospective nodes
• Three-dimensional ultrastructure links cell geometry to early myelin spatial organization

## Graphical abstract

**Figure:**
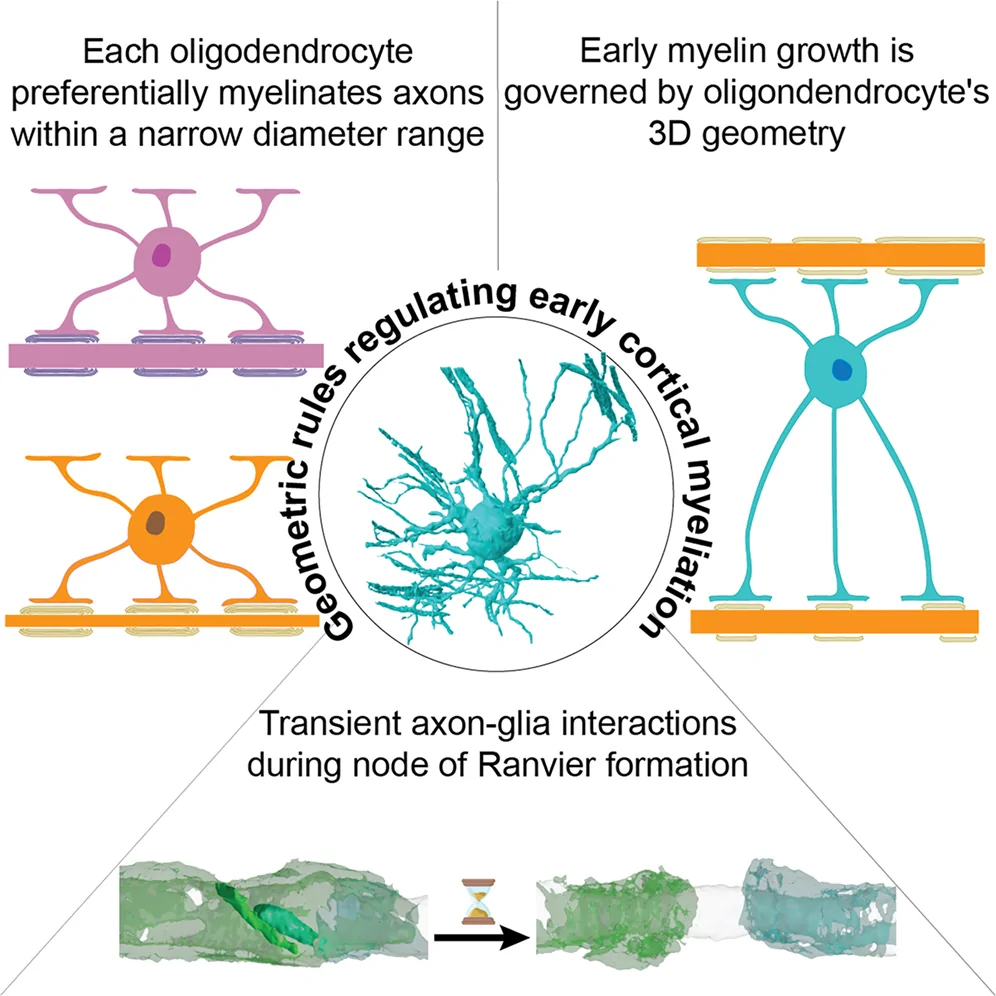

## Teaser

Neuroscience; Cell biology; Developmental biology

## Introduction

A quantitative understanding of myelin architecture, particularly the interaction between axons, oligodendrocytes (OLs) and the myelin sheath, has become central to explaining how the microstructural organization of the central nervous system (CNS) influences nerve impulse conduction, plasticity, and selective vulnerability under pathological conditions. The development of high-resolution three-dimensional (3D) imaging techniques, together with advanced automatic and semi-automatic segmentation methods, now makes it possible to systematically reconstruct the spatial relationships between axons, oligodendroglial processes, and myelin, overcoming the limits of purely qualitative descriptions.^1^^,^^2^

OLs are specialized glial cells of the CNS responsible for the formation and maintenance of the myelin sheath around axons, with a crucial role in electrical insulation, increased conduction velocity, and metabolic support to neurons. OLs actively orchestrate node of Ranvier formation in the CNS by extending cytoplasmic processes and secreting recognition molecules such as oligodendrocyte-myelin glycoprotein (OMgp), which stabilize nodal boundaries, regulate paranodal organization, and ensure proper sodium channels (NaV) clustering across multiple axons myelinated by a single cell.^3^ In fact, unlike Schwann cells (SCs) of the peripheral nervous system,^4^ a single OL can myelinate multiple segments of distinct axons, integrating neuronal-activity-dependent signals and extrinsic glial cues to dynamically modulate myelin thickness and distribution. Myelin itself is not just an insulating structure but a regulatory interface that contributes to axonal stability, metabolic exchange, and protection from inflammatory insult.^5^

In this context, accurate segmentation of axons and myelin in 3D is essential to quantify parameters such as axonal diameter, g-ratio, myelin segment length, and OL density and to relate these morphometric indices to function.^6^ Volume electron microscopy (vEM) builds on these principles by acquiring serial images through a tissue block and reconstructing them into volumetric datasets, allowing entire cells and local circuits to be visualized *in situ*. We have already used this approach for detailed mapping of glial architecture: serial block-face electron microscopy (SBF-SEM) and 3D reconstruction application have revealed the highly complex territorial organization and nano-scale process geometry of astrocytes. Furthermore, these analyses demonstrated that glial structure can be quantitatively characterized in terms of volume occupancy, surface area, and spatial relationships to synapses and vasculature, thereby directly linking morphology to functional hypotheses regarding neurotransmitter clearance, metabolic support, and neurovascular coupling.^7^^,^^8^^,^^9^

To extract such quantitative descriptors from vEM datasets, segmentation is a critical computational step in which individual cellular and subcellular elements such as axons, myelin sheaths, and glial processes are delineated and labeled throughout the volume. Segmentation can be performed manually, semi-automatically, or using machine-learning-based algorithms, and its quality directly determines the accuracy of downstream morphometric analyses, including measures of axonal caliber, myelin thickness, surface-to-volume ratios, and branching complexity. Recent tools for automatic axon and myelin segmentation have greatly expanded the feasibility of large-scale quantitative vEM studies, but their integration with complete single-cell reconstructions of glia remains relatively underdeveloped.^10^^,^^11^ Applying this combined framework of high-resolution vEM and rigorous segmentation to neurobiology is particularly powerful for dissecting glia-neuron interactions.

In the present study, we combined high-resolution SBF-SEM and 3D reconstructions to examine early-myelinating OL morphology and myelin formation at single-cell and nanoscale resolution in the developing cortex. Our analyses examine how the intrinsic geometry of individual OLs influences early myelin wrapping across multiple axonal targets, how axon caliber selection occurs at single cell level, and how adjacent OLs interact at prospective nodes of Ranvier. Together, these data provide a structural framework to investigate how cell-intrinsic spatial constraints and intercellular interactions shape early myelination and node formation in the CNS.

## Results

### Three-dimensional reconstruction defines early-myelinating oligodendrocyte morphology in the somatosensory cortex

To characterize the nanoscale morphological features that define OL development in the CNS, we manually reconstructed three myelin-forming OLs (an example of a single OL 3D reconstruction is shown in Figure 1A) and one mature cell from a high-resolution 3D SBF-SEM dataset of the somatosensory cortex (layer VI) from a P14 rat. The imaged volume measured 101.2 × 101.2 × 76.4 μm^3^, with a voxel resolution of 24.7 × 24.7 × 50 nm^3^. Reconstruction began at the soma (Figure 1B). Mature OLs soma reportedly appear more electron-dense^12^ and rich in endoplasmic reticulum (ER), with intermediate stages depending on their maturity.^13^ Primary processes extended and branched into finer processes reaching toward neighboring axons (Figure 1C). For each myelin-forming OL, we segmented 10 contacted axons and the myelin already deposited around them (Figures 1D1–1D3), whereas for the mature OL, we segmented a single fully myelinated axon as a reference (Figures 1E1–1E3). Segmentation was performed until the boundaries of the imaged volume, and axons were truncated as they approached nodes of Ranvier, consistent with the fact that individual internodes can be myelinated by different OLs (Video S1).

**Figure 1:**
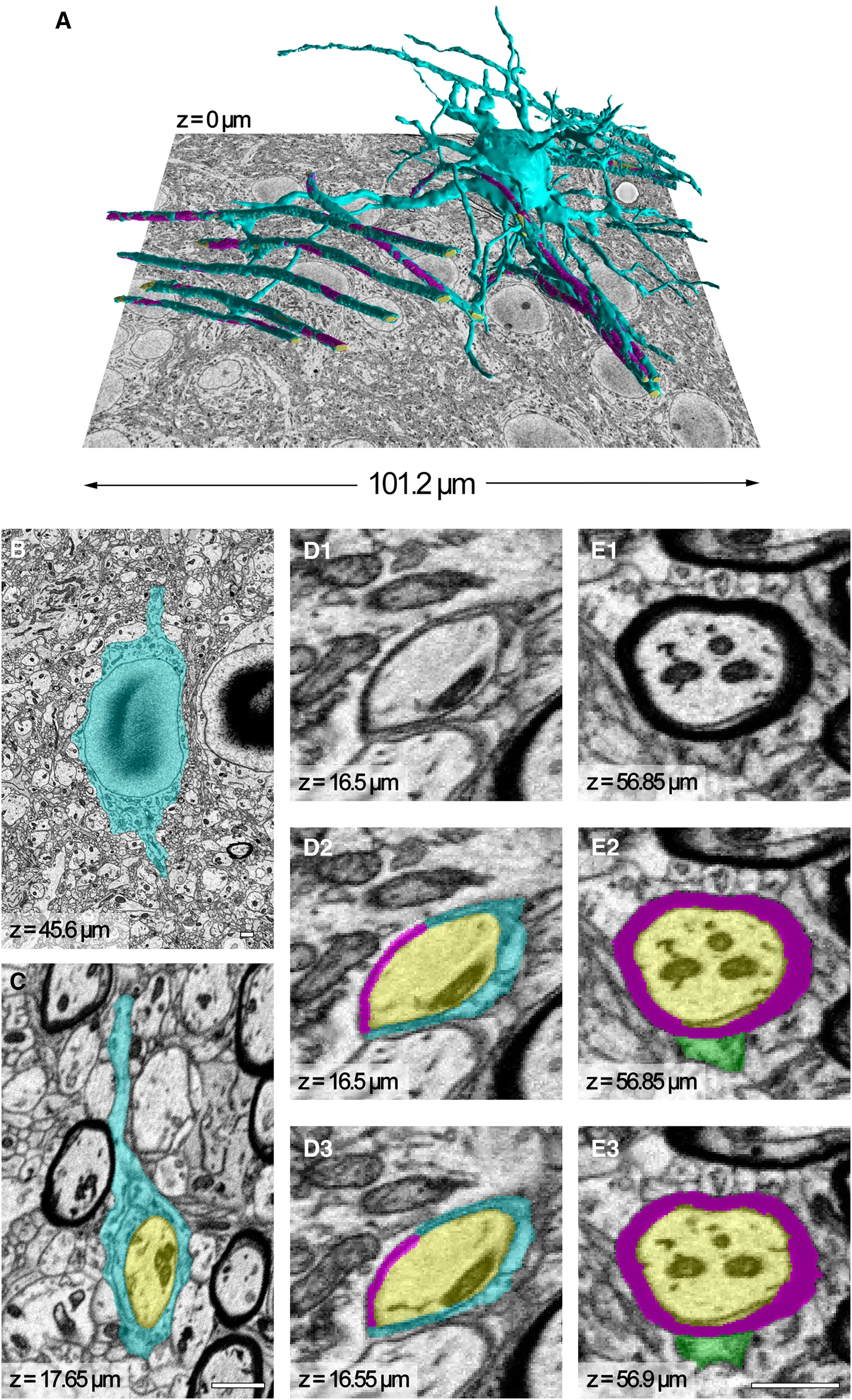
Volume imaging of OLs from layer VI somatosensory cortex (A) Serial block-face volume rendering of a myelin-forming OL (cyan) contacting 10 axons (yellow) with nascent myelin sheaths (purple). (B) Micrograph showing the OL soma body. (C) OLs are recognized by their capacity to extend cellular processes toward axons and ensheath them. (D1) Micrograph showing an incipiently myelinated axon and representative sequential EM images showing the manually delineated axonal (yellow), OL (cyan) and myelin (purple) masks used for segmentation (D2–3). (E1) Micrograph showing a mature myelinated axon and representative sequential EM images showing the manually delineated axonal (yellow), OL (green), and myelin (purple) masks used for segmentation (E2-3). Scale bars in (B), (C), and (E3) (also applicable to D1, D2, D3, E1, and E2) are 1 μm.

Video S1. Three-dimensional reconstruction of oligodendrocytes and their myelinated axonal contacts Manual three-dimensional reconstruction of three myelin-forming oligodendrocytes (OL1-cyan, OL2-grey, and OL3-ochre) and one mature OL (OL4-green) from a high-resolution SBF-SEM dataset of the somatosensory cortex (layer VI) of a P14 male rat. The imaged volume measured 101.2 × 101.2 × 76.4 μm^3^. The soma and primary processes of each OL are visible, with the latter extending and branching into finer processes toward neighboring axons. After 30″, the video zooms in on OL1, highlighting the contacted axons (yellow) and associated myelin sheaths (magenta).

### Individual oligodendrocytes synchronously initiate myelination on similar-caliber axons with distance-dependent wrapping progression

Visual inspection indicated that all axons contacted by the three myelin-forming OLs were in an active myelination stage. None appeared completely unmyelinated or fully myelinated, suggesting that, within the analyzed cells, once an OL begins depositing myelin, myelination initiates almost simultaneously across all contacted axons. To quantitatively support this observation, we measured, for each axon, both the axonal surface volume and the volume of the first myelin layer in direct contact with the axon (Figure 2A) and sequentially computed the percentage of ensheathment. The three myelin-forming OLs showed mean ± SD ensheathment values of 26.2% ± 7.3%, 20.8% ± 7.0%, and 18.7% ± 2.9%, indicating that all axons being myelinated by the same cell exhibited comparable percentages of ensheathment. Crucially, leveraging the full OLs 3D geometry available from SBF-SEM segmentation, we quantified the length of each OL process from the soma to each axonal contact and tested how this process length relates to the degree of ensheathment. In all three myelin-forming OLs, we detected a robust negative relationship between process length (i.e., distance from soma to contact site) and percentage ensheathment (OL1: R^2^ = 0.69, *p* = 0.003; OL2: R^2^ = 0.85 *p* = 0.0001; OL3: R^2^ = 0.59, *p* = 0.01), indicating that axons located farther consistently exhibited lower fractions of their surface wrapped by the first myelin layer (Figure 2B). This result suggests that the intrinsic spatial layout of an OL (specifically, the geometric distribution of its processes relative to their axonal targets) may influence the pace of early and/or faster wrapping, potentially imposing a distance-dependent constraint on initial sheath growth at distal sites. Such a spatial model extends current models of axon selection and myelin maturation, which have primarily emphasized axon-intrinsic and local microenvironmental factors, including axon diameter^14^ and neuronal-activity-dependent regulation of sheath growth and stabilization.^15^^,^^16^

**Figure 2:**
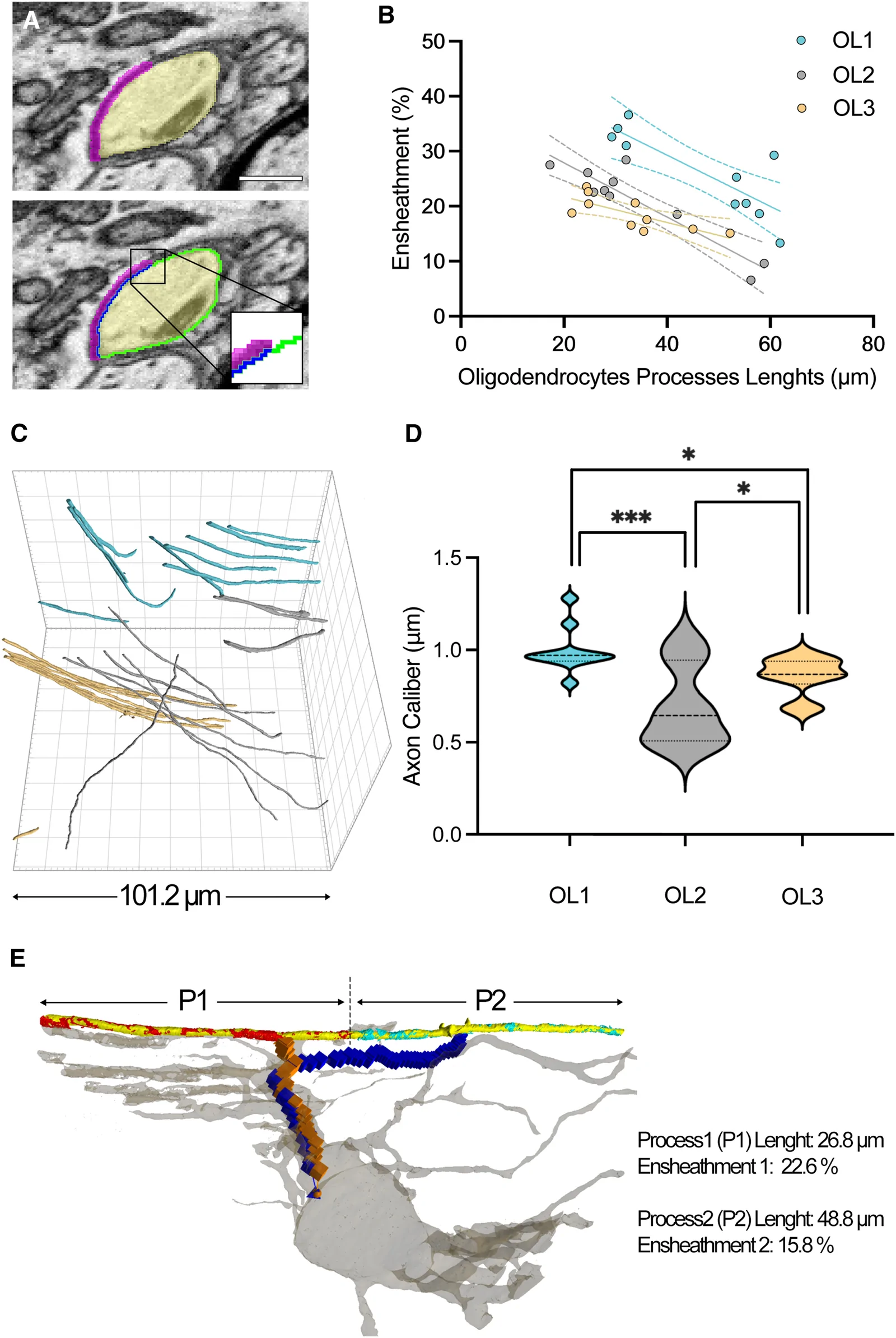
Individual OLs synchronously initiate myelination on similar-caliber axons with distance-dependent wrapping progression (A) Quantification of axonal ensheathment using Arivis pipelines. Axonal surface was approximated by generating a thin surface shell via one-pixel dilation (green). Myelin (purple) directly contacting the axon (yellow) was identified by intersecting the myelin object with the dilated axon (blue), enabling the calculation of the percentage of early ensheathment. Scale bars: 1 μm. (B) Relationship between OL process length (distance from soma to axonal contact site) and percentage of axonal ensheathment. For each OL, linear regression reveals a significant negative correlation, indicating reduced early wrapping at more distal contact sites. Each point represents one contacted axon (*n* = 10 axons per OL); solid lines show simple linear-regression fits, and dotted lines show 95% confidence intervals. Regression significance was assessed using the F-test (ANOVA). (C) Spatial distribution of axons myelinated by OL1 (cyan), OL2 (gray), and OL3 (ocher). Bounding box size: 101.2 μm (*x*, *y*) and 76.4 (*z*). (D) Violin plot analysis reveals OL-specific axon caliber preference (*n* = 10 contacted axons per OL; dashed lines indicate medians, and dotted lines indicate the first and third quartiles; ∗*p* < 0.05, ∗∗∗*p* < 0.001; Student’s *t* test). (E) Comparison of two internodes formed on the same axon (yellow) by a single OL shows that the internode reached by a more proximal to the soma process (orange) exhibits a higher percentage of early ensheathment (red), whereas the more distal internode, reached by a longer process (blue), shows reduced wrapping (cyan).

Another key observation from our reconstructions is that each myelin-forming OL showed cell-specific preferences for the calibers of the axons they targeted. Although all three analyzed cells were located within the same cortical layer and shared access to a heterogeneous axonal environment, the mean diameters of the axons they contacted and myelinated were significantly different (OL1: 1.00 ± 0.12 μm; OL2: 0.70 ± 0.21 μm; OL3: 0.86 ± 0.1 μm; multiple-comparison test: OL1-OL2 *p* < 0.001, OL1-OL3 *p* < 0.05, OL2-OL3 *p* < 0.05) (Figures 2C and 2D). To further assess whether this reflected selective targeting rather than the local availability of axon calibers, we quantified the local distribution of axonal calibers surrounding each OL-axon contact site. For each contact point, all axons with diameters ≥0.2 μm within the surrounding microenvironment (“neighboring axons”) were identified, excluding those already myelinated, as these do not represent available targets for selection. For each OL, we then calculated the absolute deviation between the diameter of each target or neighboring axon and the mean diameter of the axons contacted and myelinated by that same OL. This analysis revealed that target axons had significantly smaller deviations from the OL-specific mean diameter than neighboring axons, suggesting that each OL preferentially selected axons within a restricted caliber range (OL1: mean absolute deviation of contacted axon: 0.09 μm; neighbor: 0.53 μm; *p* < 0.0001; OL2: contacted axon: 0.13 μm; neighbor: 0.34 μm; *p* < 0.001; OL3: contacted axon: 0.09 μm; neighbor: 0.27 μm; *p* < 0.05; Figure S1). Together, these results indicate that the axons selected for myelination by each OL occupy a narrower caliber distribution than the surrounding local axonal population. This observation is consistent with non-random axonal engagement during the earliest stages of myelination and with previous *in vivo* observations that developing OLs exhibit intrinsic biases in the types of axons they choose to myelinate.^17^^,^^18^ Our data provide ultrastructural support for this selectivity at single-cell resolution and suggest it may already emerge at very early myelination stages. In contrast to this cell-specific selection for axon caliber, we found no relationship between axon diameter and the percentage of ensheathment within each OL (data not shown). Larger axons were not necessarily more advanced in wrapping, and smaller axons were not systematically less ensheathed. Thus, while axon caliber appears to be associated with initial target selection, it does not predict the degree of early myelin progression once that contact is established.

Further supporting this interpretation, we observed that the relationship between process length and early wrapping was maintained even when examining two internodes formed on the same axon. In this case, the contact reached by the shorter OL process exhibited a substantially higher percentage of ensheathment (process length = 26.8 μm; ensheathment = 22.6%), whereas the more distal contact—reached by a longer process—showed lower ensheathment (process length = 48.8 μm; ensheathment = 15.8%) (Figure 2E). The fact that two internodes on a single axon, exposed to the same axonal properties, display divergent wrapping progression solely as a function of their geometric position relative to the OL soma provides strong additional evidence that spatial constraints intrinsic to the cell contribute to regulating the pace of initial myelin growth. While this example is not intended as definitive evidence due to the absence of sufficient biological replicates, it illustrates a configuration consistent with a potential influence of OL geometry on early myelin growth dynamics.

### Three-dimensional reconstruction reveals stage-dependent oligodendrocyte morphology at prospective nodes of Ranvier

Three-dimensional modeling enables a detailed morphological analysis of myelinating glia at the node of Ranvier. We segmented and reconstructed the region encompassing the interface between two consecutive OLs contacting the same axon, spanning both nodal and paranodal domains. Analysis of two mature OLs (Figures 3A and 3B) revealed a pronounced compaction of OL cytoplasm at the paranodal borders, juxtaposed with a distinct extracellular gap corresponding to the site of node of Ranvier formation (brown asterisk in Figures 3B, 3C, and 3C′, and Video S2). Further analysis of OL morphology at the onset of myelination at prospective nodes of Ranvier showed that during early myelination, OLs display transient intra-axonal projections at future nodes. These projections were observed both at interfaces between an early and a mature myelinating OL contacting the same axon (Figure 3D) and between two early myelinating OLs (Figure 3G). In the early-mature OL configuration, the origin of the transient intra-axonal projection could be traced to the cytoplasm of the wrapping OL, emerging from a region consistent with the presumptive paranode (“type 1 infiltration”—purple arrows in Figures 3E, 3F, and 3F′) and extending across—and beyond—the region that will later develop into the node of Ranvier (“type 2 infiltration”—red asterisks in Figures 3E, 3F, and 3F′ and Video S3). When two OLs forming adjacent nascent myelin segments on the same axon were analyzed, transient intra-axonal projections arising from both OLs were detected (purple and orange arrows in Figures 3H, 3I, and 3I′). These projections converged and contacted each other at the putative site of future node formation (red asterisks in Figures 3H, 3I, and 3I′); in some instances, multiple projections extending in different directions were also observed (Video S4).

**Figure 3:**
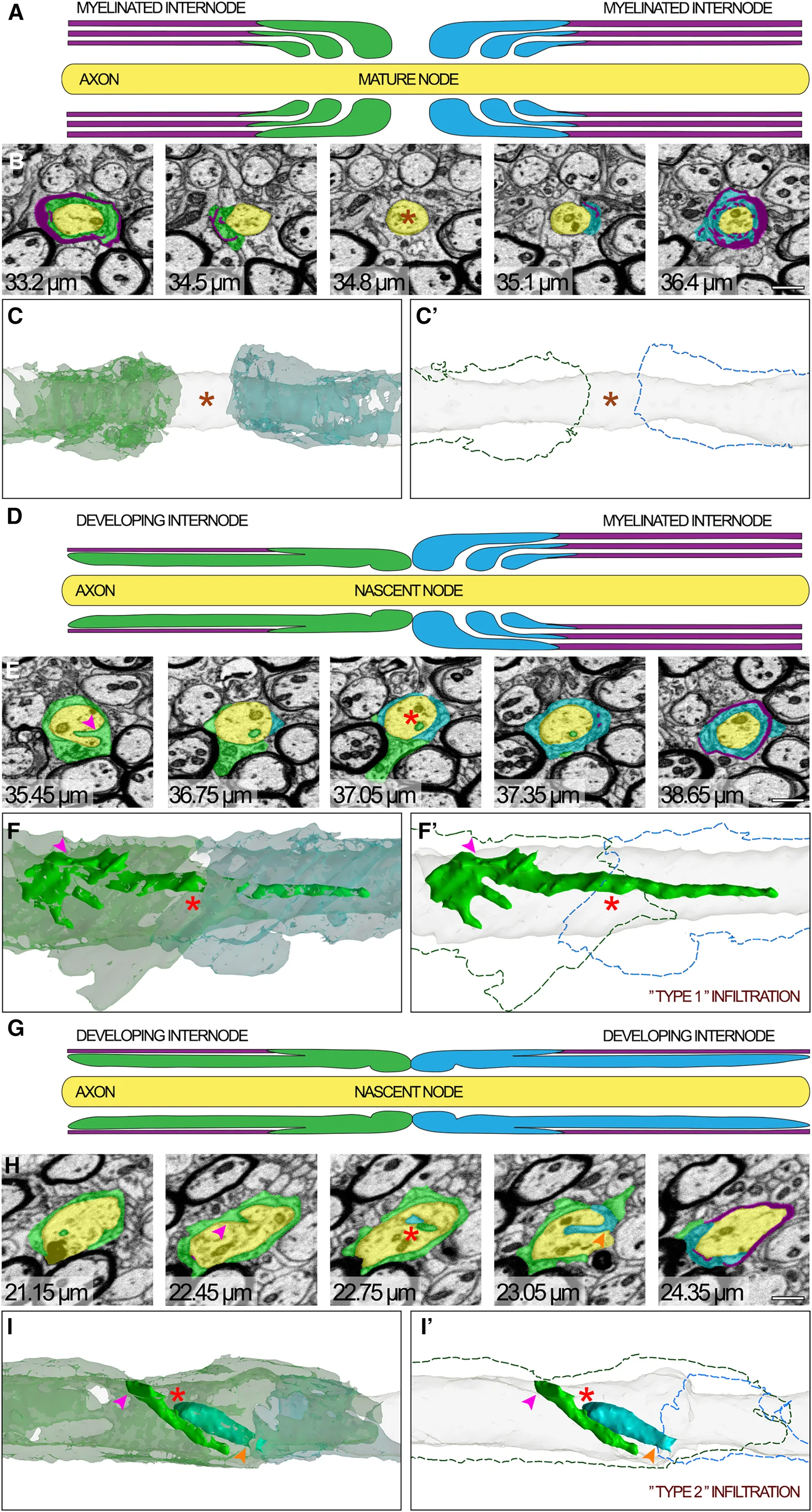
3D reconstruction reveals stage-dependent OL morphology at prospective nodes of Ranvier (A, D, and G) Schematic representations of nodal regions formed, respectively, between two fully myelinating OLs (A), between an early and a mature myelinating OL (D), and between two early myelinating OLs (G). (B, E, and H) Representative micrographs used for segmentation of the corresponding nodal and paranodal regions. (C, F, and I) Corresponding 3D reconstructions of the nodal and paranodal domains. (C′, F′, and I′) Dashed lines delineate OL cytoplasm within the paranodal-nodal interface. Scale bars in (B), (E), and (H) are 1 μm. Yellow: axon; purple: myelin; green: first OL; cyan: second OL. Brown asterisk: mature node of Ranvier site; red asterisk: nascent node of Ranvier site; purple and orange arrows: origin of the transient intra-axonal OL projection.

Video S2. Three-dimensional reconstruction of mature oligodendrocytes at the node of Ranvier Mature OL1 and OL2 are shown in green and cyan, respectively, and myelin is shown in magenta. Three-dimensional reconstruction of the interface between two consecutive mature OLs contacting the same axon, revealing a distinct extracellular gap between the two myelinating OLs and the surrounding compaction of OL cytoplasm.

Video S3. Three-dimensional reconstruction of the interface between an early myelinating and a mature oligodendrocyte Early-myelinating OL1 and mature OL2 are shown in green and cyan, respectively, and myelin is shown in magenta. The intra-axonal projection of OL1 is visible, emerging from the OL cytoplasm and extending across and beyond the region corresponding to the prospective node of Ranvier.

Video S4. Three-dimensional reconstruction of adjacent early myelinating oligodendrocytes The two early myelinating OLs are shown in green and cyan, respectively, and myelin is shown in magenta. Transient intra-axonal projections arising from both OLs are visible, extending toward and converging at the prospective node of Ranvier.

### Intra-axonal projection analysis reveals frequent oligodendrocyte projections during early node formation

To determine how frequently these transient intra-axonal projections occur during nodal formation, we extended our analysis using a complementary large-scale analytical approach. Taking advantage of the high number of myelinating axons present within the dataset, we implemented a multi-step semi-automated pipeline for the identification of myelinated axons (see STAR Methods for details). This strategy enabled the analysis of spatially restricted regions associated with nodal development without requiring full OL reconstruction. Deep-learning-based segmentation initially identified 432 axonal segments. Following manual inspection, segments belonging to the same axon were merged, yielding a total of 239 reconstructed axons. Morphometric analyses were subsequently performed on 204 axons after excluding those shorter than 20 μm, which were typically located at the borders of the imaged volume and therefore not suitable for the analysis (Figure S2A). Axons ranged from 20.05 to 113.71 μm in length, with a mean of 59.49 ± 26.27 μm (Figure S2C). Altogether, the reconstructed axons accounted for a cumulative analyzed length of 1.22 cm, and axonal diameter ranged from 0.56 to 1.39 μm, with a mean of 0.869 ± 0.121 μm (Figure S2D). These measurements provide a morphometric characterization of the axonal population included in the large-scale analysis and show that the semi-automated pipeline sampled axons spanning a broad range of diameters. Within this dataset, 84 axons exhibited at least one node of Ranvier, corresponding to 41.18% of all analyzed axons. Among these, OL process infiltration was identified in 18 developing nodes formed between a mature and a newly myelinating OL (“type 1” infiltration) and in eight developing nodes located between two early myelinating OLs (“type 2” infiltration), corresponding respectively to 20.9% and 9.5% of all identified nodes (Figure S2B). These observations indicate that transient intra-axonal projections represent a relatively frequent morphological feature during early node of Ranvier formation.

## Discussion

In recent years, increasing attention in neuroscience has focused on how three-dimensional representation of cellular structures enables the extraction of complex volumetric and topological descriptors to link morphology to functions (e.g., synaptic transmission and metabolic support).^7^^,^^9^^,^^19^^,^^20^ Building on this methodological framework, recent work on collaborative augmented reconstruction of 3D neuron morphology in mouse and human brains has shown large-scale, high-fidelity reconstructions.^21^^,^^22^^,^^23^^,^^24^^,^^25^ These studies demonstrate how quantitative 3D reconstruction can turn nanoscale ultrastructure into functional descriptors of glial contributions to circuit organization, providing a template for analogous analyses of myelinated axons. While light microscopy allows dynamic, live imaging of cells and circuits, electron microscopy provides orders-of-magnitude higher spatial resolution, at the cost of requiring fixed tissue, making it uniquely suited to resolve cells, membranes, organelles, and myelin ultrastructure. In neurobiology, this resolution is essential because neuronal and glial function depends critically on fine morphological specializations such as postsynaptic densities, presynaptic vesicles, synaptic clefts, perisynaptic astrocytic processes, and multilamellar myelin, which cannot be fully characterized with conventional light microscopy alone.^26^ Extending similar methodologies to oligodendrocytes enables single-cell-level analysis of how individual cells distribute their processes across multiple axons, how myelin internodes are arranged along axons of different calibers and at different distances from the soma, and how morphometric parameters encode the balance between conduction efficiency and metabolic demand. Such an approach positions oligodendrocytes within the broader paradigm in which glial function is inferred from high-dimensional structural descriptors extracted from volumetric EM data, thereby integrating myelination into a unified framework of glial contributions to circuit organization and function.^8^^,^^27^^,^^28^^,^^29^

Oligodendrocyte development proceeds from oligodendroglial precursors (oligodendrocyte precursor cells [OPCs]), which proliferate, migrate along specific axial domains, and subsequently differentiate into mature, myelinating oligodendrocytes. This process is controlled by a wide range of extrinsic factors (axonal signals, extracellular matrix molecules, cytokines, and growth factors) and intrinsic factors (transcriptional programs and epigenetic regulators), which orchestrate the acquisition of a myelinating phenotype and the choice of target axons. Alterations in these developmental and maturation steps, including the transition from OPCs to mature oligodendrocytes, are tightly associated with demyelinating disorders and with forms of neurodegeneration in which axonal damage and myelin loss are closely intertwined.^30^ Our three-dimensional reconstructions of early-myelinating oligodendrocytes supported the possibility that the onset of myelination occurs nearly simultaneously across multiple axonal targets and that the extent of myelin sheath progression is inversely related to the distance from the oligodendrocyte soma. This distance-dependent gradient suggests the hypothesis that the intrinsic spatial layout of individual OLs imposes a geometric constraint on initial myelin growth, complementing previously described axon-intrinsic and activity-dependent factors that regulate sheath formation.^31^ Furthermore, the compartmentalization of axon caliber at the level of single OLs indicates that each cell preferentially myelinates axons within a restricted diameter range. This observation is consistent with the possibility that cell-intrinsic programs are superimposed on general permissive thresholds, contributing to the fine-scale organization of cortical myelin. However, given the limited number of reconstructed cells and the fact that our analysis captures the tissue after axonal selection has already occurred, we cannot determine the complete pool of axons that was available to each OL during target selection and initial myelin sheath formation. However, these findings are consistent with previous work showing that local structural features contribute to the regulation of internode length, myelin thickness, and conduction efficiency.^32^

One possible interpretation of our findings is that intrinsic geometric constraints provide a structural framework upon which activity-dependent mechanisms refine axon selection and myelin stabilization. Previous studies demonstrated that neuronal activity regulates OL differentiation, axon selection, nodal clustering, and stabilization of nascent myelin sheaths through synaptic vesicle release, neurotransmitter-mediated signaling, and activity-dependent trophic support.^16^^,^^33^^,^^34^^,^^35^^,^^36^^,^^37^ *In vivo* studies further showed that electrically active axons are preferentially selected for myelination and that distinct neuronal subtypes exhibit different patterns of myelin coverage depending on their physiological activity profiles.^15^^,^^38^^,^^39^ At the same time, OLs retain intrinsic programs that regulate sheath length, spatial organization, and the general pattern of axonal engagement, suggesting that adaptive myelination mechanisms operate on top of pre-existing geometric constraints.^40^^,^^41^ Within this framework, the preferential ensheathment of axons with similar diameters by individual OLs may reflect not only intrinsic spatial organization but also shared physiological properties among neighboring axons, including coordinated firing patterns or similar developmental maturation states. Because axonal caliber, conduction properties, and neuronal activity are tightly interconnected, local populations of similarly sized axons may generate coherent activity-dependent signals that bias OL process stabilization toward specific subsets of targets. Our findings, therefore, could support a model in which intrinsic OL geometry establishes an initial structural landscape that constrains early axonal selection, while neuronal activity contributes to the selective stabilization, refinement, and plasticity of emerging myelin sheaths. Such interplay between cell-intrinsic spatial organization and adaptive activity-dependent myelination may represent an important mechanism through which OLs fine-tune conduction velocity, temporal synchronization, and circuit function during cortical development.^42^ Future studies combining optogenetic manipulation could test whether subtype-specific activity profiles during critical windows reinforce the geometric compartmentalization we observed at the single-OL level.

Oligodendrocytes play an active, instructive role in the formation of nodes of Ranvier rather than serving solely as passive insulators. Axonal compartmentalization into node, paranode, and juxtaparanode depends critically on axon-glia interactions, with oligodendrocytes forming paranodal axo-glial junctions through neurofascin-155, which establish diffusion barriers and physically segregates ion channel domains.^43^ In addition, oligodendrocyte-derived cues, including OMgp at node-paranode borders, promote NaV channels clustering and suppress aberrant membrane remodeling.^44^ During development, oligodendrocytes dynamically extend and remodel cytoplasmic processes, sensing axonal signals such as neurofascin-186 to rapidly organize nodal domains following ensheathment.^45^ A finding of our study is the transient emergence of intra-axonal projections at prospective nodes of Ranvier during early myelination. These projections arise from presumptive paranodal regions and converge between adjacent OLs on the same axon, suggesting an early, dynamic form of intercellular communication that may help establish nodal architecture. While mature internodes display compact paranodes flanking an extracellular nodal gap, these transient projections highlight that node formation is not solely a passive consequence of internode juxtaposition, but likely involves active morphological exploration and coordination between neighboring OLs.^46^ Such a mechanism may complement molecular cues, including adhesion molecules and cytoskeletal scaffolding, in organizing voltage-gated ion channel clustering and the excitable domains critical for saltatory conduction.

Our observations can be integrated within current models of node of Ranvier assembly, demonstrating that paranodal axo-glial junctions, mediated by interactions among neurofascin-155, Caspr, and contactin, are essential for the formation of membrane diffusion barriers that segregate nodal and juxtaparanodal domains and stabilize sodium channel clustering.^47^^,^^48^ In parallel, other studies showed that node formation is supported by partially overlapping mechanisms involving paranodal restriction, extracellular-matrix-mediated clustering, and cytoskeletal anchoring of NaV channel complexes.^45^^,^^49^ Furthermore, developmental and remyelination studies demonstrated that nodal assembly is a highly dynamic process characterized by progressive recruitment and stabilization of nodal proteins during early ensheathment stages.^44^^,^^50^^,^^51^ Within this framework, the transient intra-axonal projections identified in our reconstructions may represent a previously unrecognized morphological component of early nodal assembly. Rather than replacing established molecular mechanisms, these structures may act in parallel with them by facilitating localized membrane remodeling, spatial coordination between adjacent oligodendrocytes, and/or early confinement of axonal membrane domains before mature paranodal sealing is achieved. Such transient structural interactions could integrate with activity-dependent clustering of nodal proteins and therefore provide a physical scaffold that complements molecular pathways regulating sodium channel clustering, paranodal junction maturation, and stabilization of excitable axonal domains during the transition from immature heminodes to mature nodes of Ranvier.

Understanding the physiological basis of oligodendrocyte selection of specific axons, together with the role of cytoplasmic infiltration into axonal domains, is essential not only for defining the mechanisms that govern myelination during development but potentially also for elucidating remyelination processes and why myelin repair is inefficient in disease states where axonal properties or oligodendrocyte-axon interactions are altered. Defective remyelination and disruption of nodes of Ranvier in the central nervous system (CNS) are common features of several neurological disorders, leading to impaired saltatory conduction, conduction block, or slowed axonal signaling.^52^ Multiple sclerosis (MS), the prototypical CNS demyelinating disease, exemplifies nodal pathology in which immune-mediated attacks dismantle nodal and paranodal proteins such as neurofascin-186 and contactin-1, causing rapid dispersal of voltage-gated NaV channels. These alterations contribute to axonal dysfunction and degeneration, also beyond the consequences of demyelination alone.^53^ Remyelination in MS often fails to fully restore nodal architecture, contributing to persistent conduction deficits and axonal vulnerability despite partial myelin repair.^54^ While oligodendrocyte precursor cells (OPCs) can differentiate and generate thin, new myelin sheaths around demyelinated axons, these remyelinated internodes frequently exhibit disorganized clustering of nodal proteins like voltage-gated NaV, Caspr, and Kv1.1 potassium channel. Disruptions in the precise paranodal and juxtaparanodal domains, which are essential for saltatory conduction, arise from incomplete aggregation of these proteins during repair and are exacerbated by chronic inflammation, inhibitory extracellular matrix changes, and microglial dysfunction, which hinder proper myelin-axon interactions.^55^ This suboptimal nodal reformation results in inefficient signal propagation, increased energy demands on axons, and progressive neurodegeneration, even in lesions with viable OPCs.^54^

Methodologically, our work demonstrates the analytical value of systematic re-examination of existing high-resolution EM datasets, in alignment with the 3R principles (ethical guidelines for animal research emphasizing reduction, replacement, and refinement). By leveraging previously acquired volumes, we minimized the need for additional animal experiments while still extracting additional insights into OL morphology, myelination dynamics, and nodal formation. Moreover, the reuse of high-fidelity datasets enables longitudinal comparisons and the development of augmented reconstruction pipelines that can be applied across studies, thereby enhancing reproducibility and efficiency in neurobiological research. Finally, our findings also highlight several avenues for future investigation. The geometric principles uncovered here, such as the distance-dependent myelin progression, axon caliber compartmentalization, and transient nodal projections, could now be tested in pathological models, including developmental hypomyelination, traumatic brain injury, or channelopathies, to dissect the interplay between intrinsic OL programs and extrinsic axonal signals and to define the mechanistic basis of myelin plasticity and nodal stability in health and disease.

In conclusion, this study integrates single-cell, 3D ultrastructural analysis to define how intrinsic spatial constraints and intercellular interactions guide early myelination and node formation. By linking morphological descriptors to functional hypotheses, our work provides a framework for investigating both developmental myelination and its perturbation in disease, while exemplifying the scientific value of reusing existing vEM datasets.

### Limitations of the study

This study analyzes one previously acquired SBF-SEM volume from a single postnatal day 14 rat and includes complete reconstructions of three early-myelinating oligodendrocytes and one mature cell. The observed cell-to-cell patterns, therefore, cannot establish population-level generalizability. Because the tissue was fixed and sampled after axonal contact had occurred, the analysis is cross-sectional and cannot resolve temporal sequence, causal mechanisms, or the complete pool of axons available during target selection. Independent datasets, additional animals, and longitudinal or perturbational approaches will be needed to test the proposed structural model.

## Resource availability

### Lead contact

Requests for further information and resources should be directed to and will be fulfilled by the lead contact, Corrado Calì (corrado.cali@unito.it).

### Materials availability

This study did not generate new unique reagents.

### Data and code availability

• EM data are provided at https://webknossos.org/links/Ex0xZtJPNY15O9IQ.
• Analysis scripts will be made available upon reasonable request to the lead contact.
• Any additional information will be made available upon reasonable request to the lead contact.

## Acknowledgments

We thank Prof. Paola Bezzi for the generous gift of the samples and Prof. Pierre Magistretti for his valuable suggestions, financial support, and assistance. We acknowledge the Visualization Core Lab at King Abdullah University of Science and Technology (KAUST) and in particular Dr. Ronell Sicat for his expert assistance during the analysis. We thank Dr. Gabriel Herrera Lopez (KAUST) for his support for the graphical abstract. This work was supported by the “Dipartimento di Eccellenza” fundings of the Department of Neuroscience, University of Turin to C.C.

## Author contributions

C.C. and L.B. conceived the study. C.C. developed the methodology. V.C. and M.F.V.C. developed the software. V.C., M.M., M.F.V.C., and L.B. performed the validation. M.M. and L.B. conducted the formal analyses. V.C., M.M., M.F.V.C., K.K., and L.B. carried out the investigation. L.B. curated the data and prepared the visualizations. M.F.V.C. and V.C. made the graphical abstract. V.C., M.M., and L.B. wrote the original draft. L.B., M.C., and C.C. reviewed and edited the manuscript. M.C. and C.C. supervised the study. C.C. administered the project and acquired funding. All authors read and approved the final version of the manuscript.

## Declaration of interests

V.C. has a PhD scholarship co-financed by Zeiss. L.B. is an external collaborator of IQVIA inc.

## STAR★Methods

### Key resources table

**Table:** 

REAGENT or RESOURCE	SOURCE	IDENTIFIER
**Deposited data**		

Volume electron microscopy dataset	webKnossos	https://webknossos.org/links/Ex0xZtJPNY15O9IQ

**Experimental models: Organisms/strains**		

P14 Rat, Sprague-Dawley, Male	University of Lausanne	N/A

**Software and algorithms**		

Python 3.12	Python	RRID:SCR_008394 https://www.python.org/
GraphPad Prism 9.5.1	GraphPad software	RRID:SCR_002798 https://www.graphpad.com/
Fiji (ImageJ)	Fiji	RRID:SCR_002285 https://fiji.sc/
Arivis Pro 4.3.0	ZEISS	N/A https://www.zeiss.com/microscopy/en/products/software/advanced-image-analysis.html#arivis-pro
WebKnossos	WebKnossos	N/A https://home.webknossos.org/
Avizo software	Thermo Fisher Scientific	N/A https://www.thermofisher.com/it/en/home/electron-microscopy/products/software-em-3d-vis/avizo-software.html
Napari	Napari	N/A https://napari.org/stable/
Fine-tuned Panoptic DeepLab myelin segmentation model	This paper	Available upon request

### Experimental model and study participant details

This study reanalyzed a previously published SBF-SEM dataset acquired from layer VI of the somatosensory cortex of a Sprague-Dawley male rat at postnatal day 14. The brain tissue was generously provided by Prof. Paola Bezzi (University of Lausanne, Switzerland), and collected in accordance with the Swiss Laws for the protection of animals.^7^ No new animals were used for the present secondary analysis.

### Method details

#### Sample preparation and imaging

The original volume electron microscopy dataset used in this work has been previously described and published in Calì et al., 2019.^7^ A P14 rat was deeply anesthetized with an intraperitoneal injection of 150 mg/kg pentobarbital sodium and subsequently transcardially perfused with fixative solution (2% paraformaldehyde and 2.5% glutaraldehyde in 0.1 M phosphate buffer - total volume of 200 mL). 100 μm coronal sections were cut using a vibratome and stored in 0.1 M phosphate buffer until embedding. Samples were prepared as follows: sections were washed in cacodylate buffer, followed by a post-fixation with ice-cold reduced osmium tetroxide, thiocarbohydrazide solution and osmium tetroxide. Sections were then incubated in uranyl acetate, followed by *en bloc* Walton’s lead aspartate staining. Finally, the sections were embedded in Durcupan resin. Layer VI of the somatosensory cortex was selected, mounted on an aluminum pin and the block was trimmed prior to application of silver paint to minimize specimen charging.^56^ Tissue was imaged using backscattered electrons with a serial block-face scanning electron microscope (3View), acquiring a total volume of 101.2 × 101.2 × 76.4 μm^3^, with resolution of 24.7 × 24.7 × 50 nm^3^.

#### 3D reconstruction, rendering and analysis

OL, myelin, and axonal areas were manually segmented using the TrackEM2 plugin in Fiji software, then imported and rendered in Arivis Pro 4.3.0 (Carl Zeiss AG, Oberkochen, Germany), assigning a specific tag to each structure. During segmentation, we ensured that axon and myelin masks were precisely apposed (without overlapping pixels) so that the axon–myelin interface accurately reflected the underlying ultrastructure. To improve reconstruction accuracy and consistency, each segmented cell was independently reviewed by an independent investigator. Ambiguous regions or potential tracing inconsistencies were jointly discussed and corrected when necessary. Finally, an additional third reviewer performed a global quality-control assessment across all reconstructed cells to ensure overall coherence and continuity of the final segmentations.

In order to quantify the percentage of myelin ensheathment for each axon, two custom Arivis Pro analysis pipelines were implemented. The first pipeline was used to calculate axonal surface area: each axon object was imported into the pipeline and processed with the “Segment Morphology” operation, applying a dilation with pixel parameters set to 1, using the *Box* shape, and enabling the *Perform plane-wise* option. The dilated object was then subtracted from the original axon using the “Object Math” operation, and the resulting object was exported for further analysis. This procedure effectively generated a thin surface shell surrounding the axon, which served as an approximation of the axonal surface for subsequent quantification. The second pipeline was developed to quantify the portion of myelin in direct contact with the axon. For each axon-myelin pair, the same *Segment Morphology* parameters were applied to the axon. The intersection between the myelin object and the dilated axon object was computed using the *Object Math* operation, generating a new object corresponding to the myelin directly contacting the axonal surface. The percentage of ensheathment was calculated by dividing the volume of the myelin in direct contact by the volume of the axonal surface. Since Arivis Pro treats segmented elements as volumetric 3D objects, it enables volume-based analyses but does not provide direct length measurements. Therefore, in order to determine the length of OL processes and axons, a 3D polyline was manually created for each segment, following their full 3D trajectory. To calculate axonal diameter, axons were approximated as cylinders, and the diameter was derived from the measured volume and length of each axonal segment.

To test whether myelin-forming OLs exhibit preferences for specific axon diameters, we quantified the local distribution of axonal calibers surrounding each OL–axon contact site. Specifically, all axons within the local microenvironment (“neighboring axons”) with diameters ≥0.2 μm were considered, as this value represents the approximate minimum axon caliber reported to support myelination in the CNS.^57^ A cubic subvolume (10 μm per side) centered on each OL–axon contact point was extracted from the dataset. Within each subvolume, axons were segmented using the pre-trained deep learning model “Neuron Segmentation” implemented in WebKnossos^58^ (Scalable minds UG, Potsdam, Germany). The resulting segmented axonal surfaces—both contacted (target) and neighboring axons—were then imported into Arivis Pro for morphometric analysis. Axon diameters were computed for each individual axon using the same procedure described above.

For the computation of nodal structures, we implemented a multi-step image-processing pipeline as follows: (*i*) First, we generated a deep-learning model for the automatic segmentation of myelin structures surrounding axons. The rationale for focusing on myelin rather than directly segmenting axons derives from the highly electron-dense appearance of myelin rings in EM images, which makes these structures particularly suitable for automated recognition. A representative subset of the dataset was selected by defining regions of interest (ROIs) of 512 × 512 pixels, within which representative myelin structures were manually segmented. These manual annotations were generated using Avizo software (Thermo Fisher Scientific) using a Wacom Cintiq pen and tablet interface. (*ii*) Myelin segmentation was subsequently performed using a fine-tuned Panoptic DeepLab model implemented through the Empanada plugin within the Napari framework. The model was trained on the manually annotated regions described above and subsequently applied to the entire image stack to generate an automatic myelin segmentation mask for the full volume. The predicted myelin segmentation was then imported into Avizo software as label/mask data for post-processing and downstream derivation of axonal masks. Small isolated regions containing fewer than 200,000 voxels were removed using the “Remove Islands” module. To improve the continuity of incompletely closed myelin sheaths in two-dimensional sections, sequential “Grow” and “Shrink” morphological operations were applied to the myelin masks. (*iii*) Once the myelin mask had been generated, the “Fill Holes” operation was applied to obtain the enclosed axonal areas by filling lumen regions surrounded by myelin in each section. (*iv*) Finally, the axonal mask was extracted by computing the set difference between the filled label data (representing myelin together with enclosed interiors) and the myelin-only labels. This operation yielded a final label set corresponding to axonal volumes. (*v*) The axonal masks subsequently underwent expert proofreading combined with targeted manual corrections. Particular attention was devoted to axons that were not fully enclosed by myelin and were therefore incompletely captured by the hole-filling procedure. In these cases, axons were manually segmented every 10 slices through sparse annotations, followed by interpolation between annotated sections to generate a continuous three-dimensional segmentation. (*vi*) Following proofreading and manual correction, volumetric measurements for each axon were computed using the “Label Analysis” module in Avizo software. Subsequently, the “Centerline Tree” module was used to compute axonal centerlines, enabling quantification of axonal curvilinear length from the reconstructed three-dimensional trajectories, which was used as an estimate of axonal path length. To estimate axonal diameter, each axon was approximated as a perfect cylinder, and the diameter was derived as previously described. (*vii*) During manual proofreading and correction of axonal segmentations, each reconstructed axon was systematically inspected for regions displaying infiltration of oligodendrocyte processes into the axonal domain. Whenever an infiltration event was identified, it was annotated and classified according to the apparent maturation state of the adjacent oligodendrocytes (Figure 3). Infiltrations associated with at least one near-mature OL were classified as “Type 1,” whereas infiltrations occurring between two early myelinating OLs were classified as “Type 2”. Nodes of Ranvier were also identified and quantified for each analyzed axon during the same inspection procedure. Both node counts and infiltration classifications were recorded on a per-axon basis for subsequent analyses.

### Quantification and statistical analysis

#### Statistical analysis

Linear regression was performed for each OL to examine the relationships between the percentage of myelin ensheathment, as well as the length of its processes and the axon diameter. For each cell, paired measurements across all analyzed segments were used to compute the regression slope, coefficient of determination (R^2^), and the *p*-value of the F-test (ANOVA) evaluating the overall significance of the regression model. Residuals were inspected for normality using the Shapiro–Wilk test to ensure that the assumptions of linear regression were satisfied. To compare the diameter of contacted axons with the distribution of neighboring axons, statistical significance was assessed using the Mann–Whitney U test. All analyses were conducted using Python 3.12 or GraphPad Prism 9.5.1. Unless otherwise stated, n denotes an individual axon or myelinated segment. Linear regressions used *n* = 10 contacted axons for each early-myelinating OL. Pairwise differences in contacted-axon diameter among OL1, OL2, and OL3 were evaluated with Student’s *t* test as indicated in Figure 2D. Comparisons between contacted and neighboring axons used the Mann–Whitney U test. Large-scale morphometry included *n* = 204 axons, and infiltration frequencies were calculated across *n* = 84 identified nodes. Summary values reported in the text are mean ± SD. Violin plots show individual observations, medians (dashed lines), and the first and third quartiles (dotted lines). Exact *p* values are reported where available; *p* < 0.05 was considered statistically significant. In Figures 2 and S1, statistical significance is noted as follows: *p* < 0.05 (∗), *p* < 0.001 (∗∗∗), and *p* < 0.0001 (∗∗∗∗).
