# Supplementary material for: Volume electron microscopy reveals oligodendrocyte axon selection patterns and spatial assembly of myelin and nodes
Source: iScience. 2026 Oct 7;29(11):117773. doi: 10.1016/j.isci.2026.117773 (PMC13676743; doi:10.1016/j.isci.2026.117773)
Supplement: Supplementary file 1 [file mmc1.pdf]

iScience, Volume *29*

Supplemental information

Volume electron microscopy reveals oligodendrocyte axon selection patterns and spatial assembly of myelin and nodes

Martina Monaco, Vanessa Chiappini, Maria Fernanda Veloz Castillo, Kalpana Kare, Marinella Clerico, Luca Bartesaghi, and Corrado Calì

SUPPLEMENTARY MATERIAL

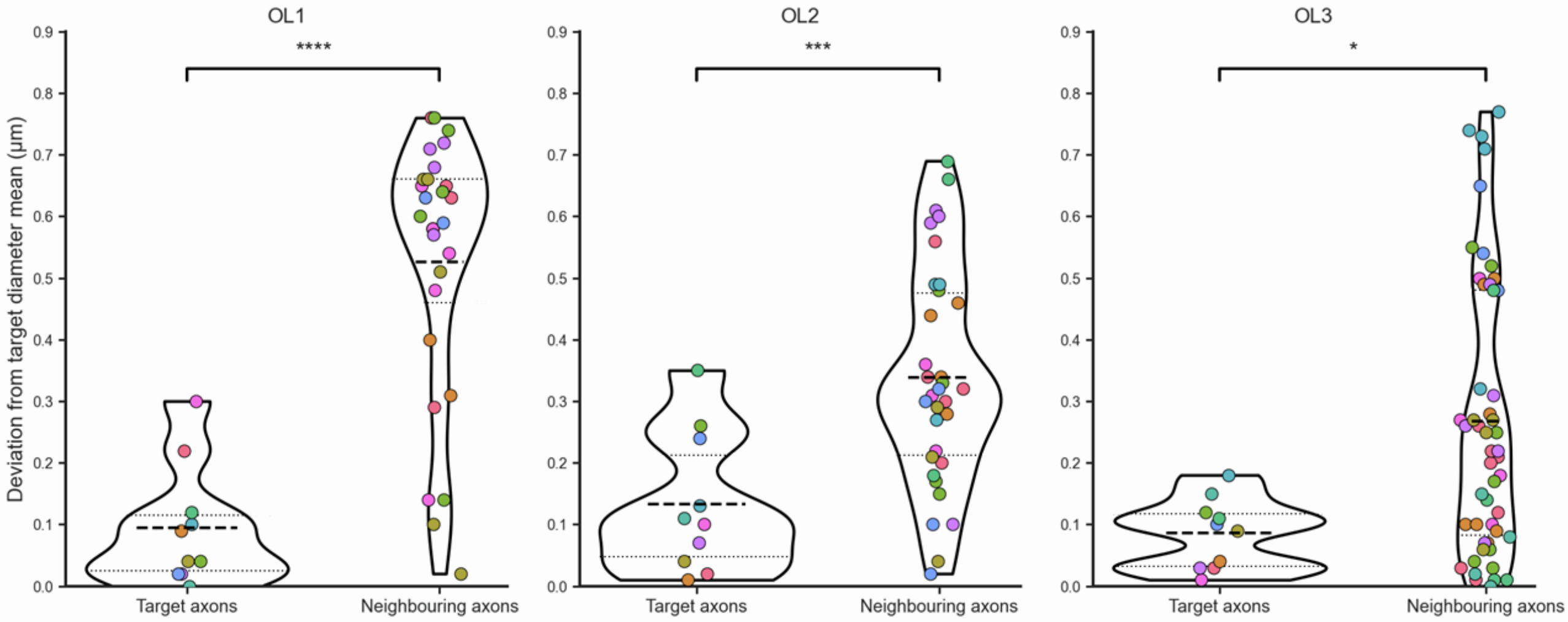

Figure S1

Analysis of OL-specific axon caliber selectivity. Violin plots show, for each OL (OL1, OL2 and OL3), the absolute deviation of the diameter of contacted target axons and neighbouring axons from the OL-specific mean diameter of the target axons caliber. Target axons consistently displayed significantly lower absolute deviations compared to neighbouring axons, consistent with the possibility that individual OLs preferentially engage axons within a relatively restricted caliber range during the early stages of myelination (OL1: target axon mean absolute deviation = 0.09 µm, neighbouring axons = 0.53 µm, **** $p < 0.0001$; OL2: target axon = 0.13 µm, neighbouring axons = 0.34 µm, *** $p < 0.001$; OL3: target axon = 0.09 µm, neighbouring axons = 0.27 µm, * $p < 0.05$). Colored dots represent individual target axons and correspond to the same-colored neighbouring axon population. Target axons: n = 10 per OL. Neighbouring axons OL1: n = 28; OL2: n = 36; OL3: n = 50. Dashed lines indicate medians and dotted lines indicate the first and third quartiles. Significance was assessed using the Mann–Whitney U test (* $p < 0.05$, *** $p < 0.001$, **** $p < 0.0001$).

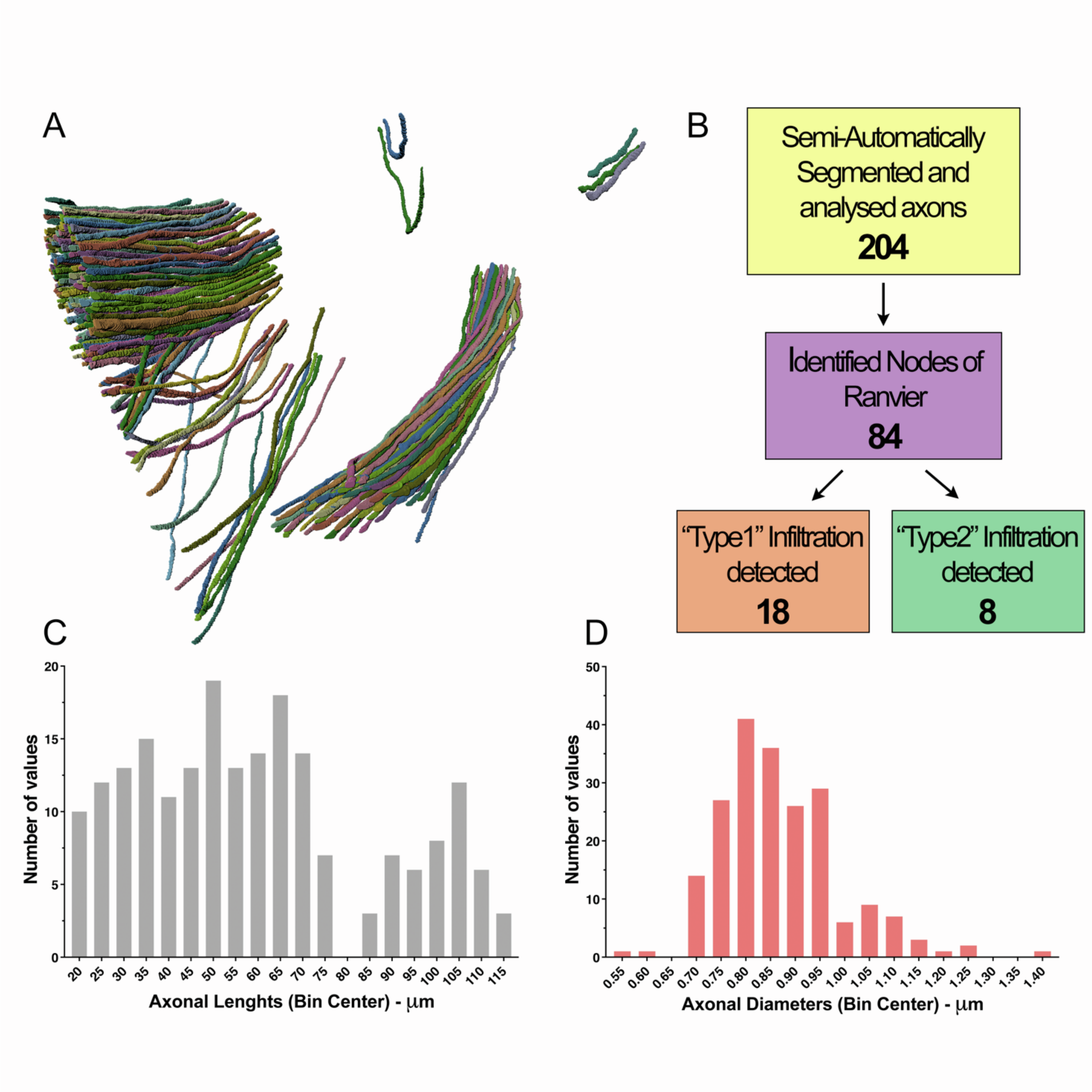

Figure S2

Intra-axonal projection analysis. (A) Spatial distribution of the semi-automatically segmented myelinated axons rendered in randomly assigned distinct colors. (B) Schematic representation of the analysis showing that, among 204 analyzed axons, 84 Nodes of Ranvier were identified, including 18 with "Type 1" infiltrations and 8 with "Type 2" infiltrations. (C) Frequency distribution histogram of the axonal lengths obtained from semi-automatically segmented axons. (D) Frequency distribution histogram of axonal diameters.
